# Bioinformatics Analysis of the Molecular Mechanism of Aging on Fracture Healing

**DOI:** 10.1155/2018/7530653

**Published:** 2018-12-16

**Authors:** Shu-Jie Zhao, Fan-Qi Kong, Jin Fan, Ying Chen, Shuai Zhou, Ming-Xin Xue, Guo-Yong Yin

**Affiliations:** ^1^Department of Orthopedics, The First Affiliated Hospital of Nanjing Medical University, Nanjing, Jiangsu 210029, China; ^2^Affiliated Hospital of Nanjing University of TCM, Nanjing, Jiangsu 210029, China; ^3^Department of Massage, The First Affiliated Hospital of Nanjing Medical University, Nanjing, Jiangsu 210029, China

## Abstract

Increasing age negatively affects different phases of bone fracture healing. The present study aimed to explore underlying mechanisms related to bone fracture repair in the elderly. GSE17825 public transcriptome data from the Gene Expression Omnibus database were used for analysis. First, raw data were normalized and differentially expressed genes (DEGs) were identified. Next, Kyoto Encyclopedia of Genes and Genomes (KEGG) and Gene Ontology (GO) analyses were implemented to evaluate pathways and DEGs. A protein–protein interaction (PPI) network was then constructed. A total of 726, 861, and 432 DEGs were identified between the young and elderly individuals at 1, 3, and 5 days after fracture, respectively. The results of GO, KEGG, and PPI network analyses suggested that the inflammatory response, Wnt signaling pathway, vascularization-associated processes, and synaptic-related functions of the identified DEGs are markedly enriched, which may account for delayed fracture healing in the elderly. These findings provide valuable clues for investigating the effects of aging on fracture healing but should be validated through further experiments.

## 1. Introduction

The elderly population has been steadily growing worldwide, and persons aged* ⩾*65 years are projected to comprise 16.9% of the American population by 2030 [1, 2]. Bone fractures are a common orthopedic problem, with a residual lifetime fracture risk in a 60-year-old person reported to be 29% in males and 56% in females, and pose an extensive medical and socioeconomic burden [3, 4]. The elderly are at a higher risk of delayed healing or non-union after bone fracture, which can have severe and systemic consequences in this age group [5, 6]. Taking into account this reality, a better understanding of age-related effects on fracture healing is critical for the development and optimization of effective therapeutic treatments.

Fracture healing is a complex but well-orchestrated process that can be temporally divided into three partially overlapping processes, namely, inflammation, repair, and remodeling [7–9]. In the initial phase, temporal control of the inflammatory response is necessary to initiate healing [10]. The inflammatory phase involves stimulating angiogenesis, attracting mesenchymal stem cells (MSCs), and promoting their differentiation, as well as enhancing extracellular matrix synthesis [11].

However, changes in the inflammatory system occur with age. In older animal models, prolonged inflammation results in delayed chondrogenesis and smaller callus size [12, 13]. Age-related decline of MSC quantity and quality may negatively affect the ability of these cells to support fracture healing, as demonstrated in humans and animal models [14, 15]. Delayed fracture healing is also associated with reduced growth factor levels and impaired angiogenesis, which are also characteristic of advanced age [16–18]. Such age-related changes are accompanied by changes in the regulation of critical molecular events involved in fracture healing, particularly those occurring early in the bone repair process [19, 20]. However, molecular changes that occur in a temporal manner are yet to be elucidated.

In this study, transcriptional analysis of GSE17825 was used to illustrate molecular mechanisms by which age affects fracture healing. Specifically, differentially expressed genes (DEGs) at three different time-points, namely, 1, 3, and 5 days after fracture, were evaluated by pathway and functional enrichment analysis. A protein–protein interaction (PPI) network was then constructed using these DEGs. These analyses revealed several molecular mechanisms that may contribute to age-related changes in fracture healing.

## 2. Materials and Methods

### 2.1. Transcriptome Data

Transcriptome profiles of GSE17825, which was submitted by Liang et al., were obtained from the National Centre of Biotechnology Information (NCBI) Gene Expression Omnibus database (GEO, http://www.ncbi.nlm.nih.gov/geo/). GSE17825, which comprises a total of 18 chips, including nine old group (8-month-old mice) and nine young group (6-week-old mice) samples, was based on the platform of the PL1261 Affymetrix Mouse Genome 430 2.0 Array. For each group, fracture calluses were dissected and total RNA was isolated at each of the three time-points (n = 3 per time-point).

### 2.2. Data Preprocessing

The robust multichip average algorithm from the Oligo package (version 1.42.0, http://www.bioconductor.org) was used to preprocess non-normalized raw data by background correction normalization, probe summary, and log2 transformation [21]. Probe identification numbers (IDs) were then converted into gene symbols, and if different probes were annotated to the same gene, then the average value served as the gene's expression level.

### 2.3. Identification and Analysis of DEGs

In this study, several paired groups were compared as follows: (i) old vs. young group at 1 day; (ii) old vs. young group at 3 days; and (iii) old vs. young group at 5 days. Student's t-test was used for comparisons. Genes with fold changes > 2.0 and P < 0.05 were considered DEGs.

### 2.4. Pathway and Functional Enrichment Analysis

The Database for Annotation, Visualization, and Integrated Discovery (DAVID 6.8; http://david.abcc.ncifcrf.gov) can provide functional annotation of enormous quantities of genes derived from different genomic resources. In this study, DAVID was used to conduct Kyoto Encyclopedia of Genes and Genomes (KEGG) pathway and Gene Ontology (GO) analyses to determine significant DEGs [22]. GO terms were determined as categories of biological processes (BPs), and P < 0.05 was defined as the cut-off criterion.

### 2.5. PPI Network Construction

The Search Tool for the Retrieval of Interacting Genes/Proteins (STRING) database (http://www.string-db.org) was used to evaluate potential protein–protein interactions. Cytoscape is an open-source tool for network visualization of genes, proteins, and other types of BPs [23]. The default confidence cut-off of 400 was used, as previously described [24]. Experimentally validated interactions, as revealed by solid lines, were included, and single nodes without interactions were excluded [24].

## 3. Results

### 3.1. Data Preprocessing and DEG Screening

**Figure 1:**
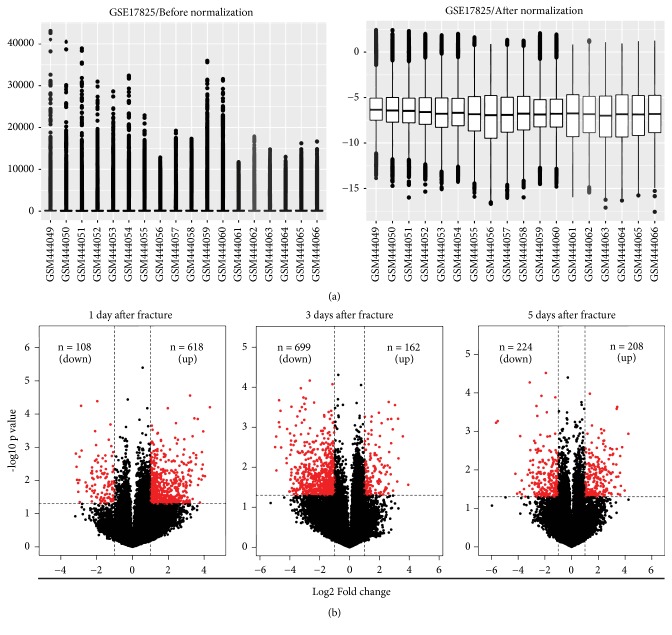
(a) Data normalization of differentially expressed genes (DEGs). Box plots of gene expression in the old and young groups at the fracture site (left panel) before and (right panel) after normalization. (b) Volcano plots at 1, 3, and 5 days of the old vs. young groups.

**Figure 2:**
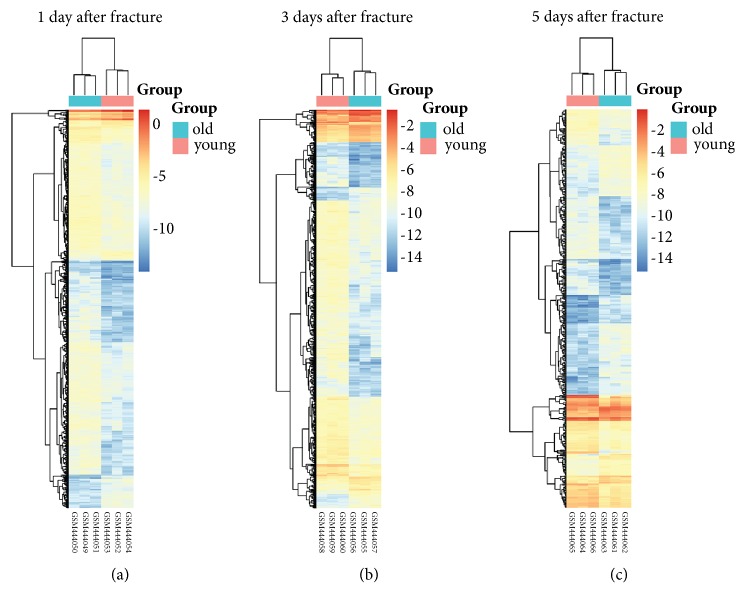
Heat maps of the genes at 1 (a), 3 (b), and 5 (c) days after SCI in the old vs. young groups. Horizontal axis represents each sample, and the vertical axis represents each gene. Blue and red colors represent low and high expression values, respectively.

Box plots of GSE17825 value distribution before and after data normalization were generated (Figure 1(a)). Data from each sample were generally normalized and cross-comparable following normalization (Figure 1(a)). DEGs between the old and young groups at the three time-points were then analyzed. A total of 618, 162, and 208 DEGs were upregulated and 108, 699, and 224 DEGs were downregulated at 1, 3, and 5 days after fracture, respectively (Figure 1(b)); volcano plots were used to visualize the identified DEGs (Figure 1(b)). Finally, heat maps of gene expression values were constructed with color patterns indicating the variability in gene expression between the old and young groups after fracture (Figure 2).

### 3.2. KEGG Pathway and GO Enrichment Analysis of DEGs

**Figure 3:**
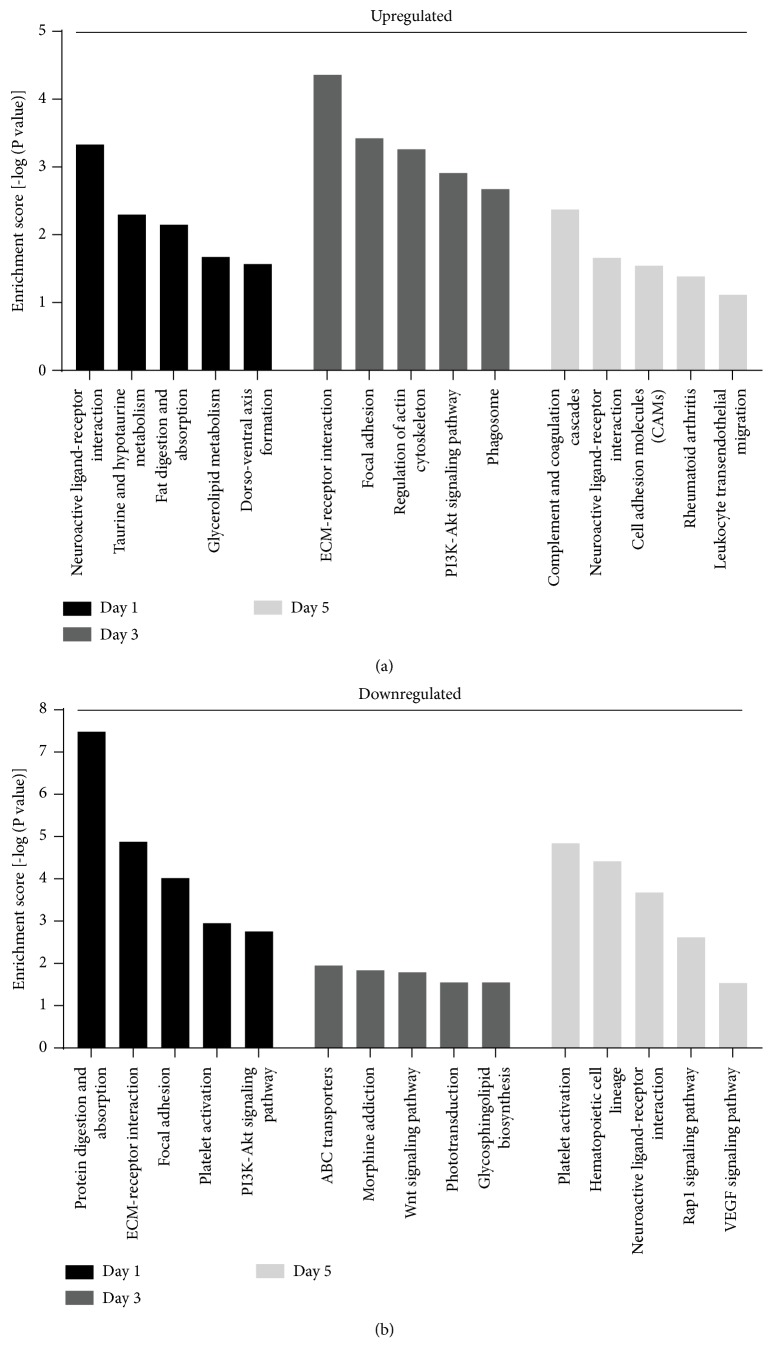
Kyoto Encyclopedia of Genes and Genomes pathways of (a) upregulated and (b) downregulated DEGs at three time-points (1, 3, and 5 days) following bone fracture.

The five most enriched KEGG pathways of up- and downregulated DEGs at each time-point (1, 3, and 5 days) are presented in Figures 3(a) and 3(b), respectively. At 1 day, the upregulated DEGs were enriched in pathways including neuroactive ligand–receptor interaction (P = 4.65 × 10^−4^), taurine and hypotaurine metabolism (P = 5.02 × 10^−3^), fat digestion and absorption (P = 7.08 × 10^−3^), glycerolipid metabolism (P = 2.13 × 10^−2^), and dorsoventral axis formation (P = 2.69 × 10^−2^). At 3 days, the upregulated DEGs were primarily involved in ECM–receptor interaction (P = 4.38 × 10^−5^), focal adhesion (P = 3.76 × 10^−4^), regulation of actin cytoskeleton (P = 5.50 × 10^−4^), PI3K-Akt signaling pathway (P = 1.23 × 10^−3^), and phagosome (P = 2.13 × 10^−3^). Finally, at 5 days after fracture, these were involved in complement and coagulation cascades (P = 4.25 × 10^−3^), neuroactive ligand–receptor interaction (P = 2.19 × 10^−2^), cell adhesion molecules (P = 2.86 × 10^−2^), rheumatoid arthritis (P = 4.11 × 10^−2^), and leukocyte transendothelial migration (P = 7.69 × 10^−2^).

At 1 day, the downregulated DEGs were involved in protein digestion and absorption (P = 3.28 × 10^−8^), ECM–receptor interaction (P = 1.33 × 10^−5^), focal adhesion (P = 9.59 × 10^−5^), platelet activation (P = 1.13 × 10^−3^), and PI3K-Akt signaling pathway (P = 1.76 × 10^−3^). At 3 days, these were primarily enriched in pathways related to ABC transporters (P = 1.12 × 10^−2^), morphine addiction (P = 1.45 × 10^−2^), Wnt signaling pathway (P = 1.61 × 10^−2^), phototransduction (P = 2.82 × 10^−2^), and glycosphingolipid biosynthesis: lacto and neolacto series (P = 2.82 × 10^−2^). Finally, at 5 days after fracture, these were enriched in vascularization-associated pathways including platelet activation (P = 1.43 × 10^−5^), hematopoietic cell lineage (P = 3.86 × 10^−5^), and VEGF signaling pathway (P = 2.89 × 10^−2^).

The 10 most significantly enriched GO terms (BPs) for the identified DEGs are listed in Table 1. At 1 day after fracture, DEGs were involved in development-associated BPs, such as skeletal system development (P = 3.32 × 10^−6^), tissue development (P = 5.61 × 10^−6^), negative regulation of multicellular organismal process (P = 6.69 × 10^−6^), negative regulation of cell development (P = 3.32 × 10^−6^), and trabecular formation (P = 1.04 × 10^−4^). At 3 days after fracture, these were the most enriched in antigen processing and peptide or polysaccharide antigen presentation via MHC class II (P = 5.58 × 10^−7^). At 5 days after fracture, these were predominantly associated with synaptic-related functions, such as anterograde trans-synaptic signaling (P = 5.80 × 10^−7^), trans-synaptic signaling (P = 5.98 × 10^−7^), synaptic signaling (P = 7.67 × 10^−7^), neuron–neuron synaptic transmission (P = 1.00 × 10^−5^), and chemical synaptic transmission (P = 4.16 × 10^−5^).

### 3.3. PPI Network Construction and Functional Module Analysis

**Figure 4:**
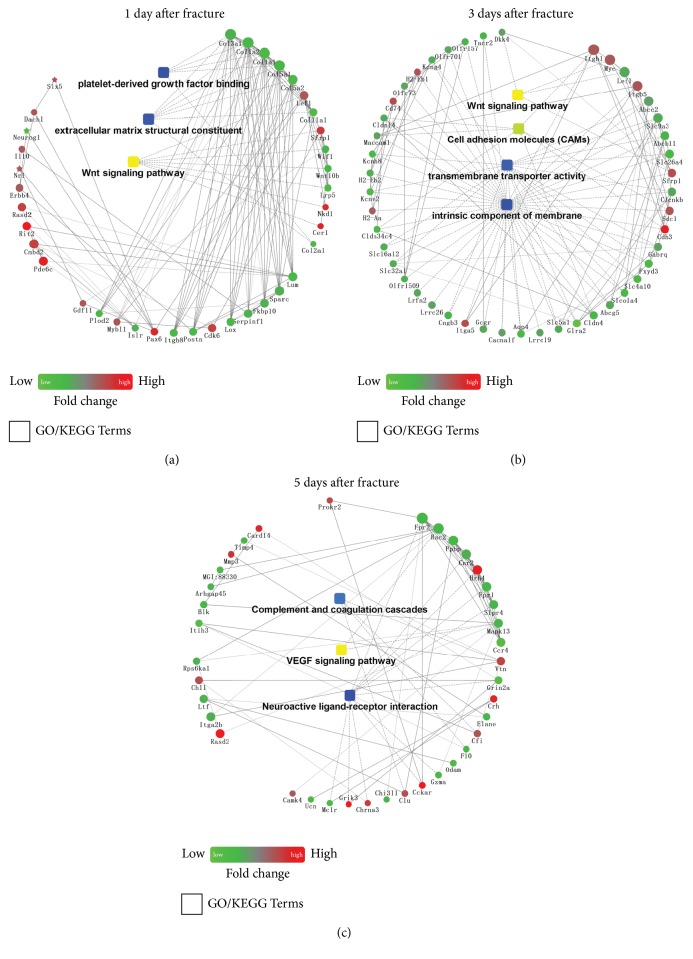
PPI networks based on DEGs. The gradient colors from green to red indicate increasing P values. Circular nodes indicate genes/proteins. Rectangular nodes indicate a biological process or a KEGG pathway. DEGs, differentially expressed genes; PPI, protein–protein interaction; KEGG, Kyoto Encyclopedia of Genes and Genomes.

PPI network analysis was performed using Cytoscape software. Results revealed that, at 1 day after fracture, DEGs were enriched in platelet-derived growth factors binding, extracellular matrix structural constituent, and the Wnt signaling pathway (Figure 4(a)). At 3 days, DEGs were involved in the Wnt signaling pathway, cell adhesion molecules (CAMs), transmembrane transporter activity, and intrinsic component of membrane (Figure 4(b)). Finally, at 5 days after fracture, DEGs were enriched in complement and coagulation cascades, VEGF signaling pathway, and neuroactive ligand–receptor interaction (Figure 4(c)).

## 4. Discussion

Fracture healing involves multiple biological phases that are characterized by both anabolic and catabolic processes [7]. While aging has a significant impact on skeletal physiology, precise mechanisms that delay fracture healing in the elderly remain unclear [25]. In the present study, bioinformatics analysis was used to determine molecular events and pathological states that occurred in the early phases of fracture healing at different organismal ages. GSE17825 database transcriptome data were collected from fracture calluses of old and young C57BL6 mice (8-month-old and 6-week-old mice) at 1, 3, and 5 days after fracture. Analysis of gene expression at these three time-points revealed DEGs between the subject groups, and KEGG pathway and GO enrichment analyses were subsequently performed. A PPI network was then constructed to further analyze molecular processes underlying fracture healing. The findings of these analyses may contribute to a better understanding of the effects of aging on fracture healing.

Because inflammation is a critical step in fracture healing, any disruption of this process can negatively affect overall healing [9]. Our GO enrichment analyses revealed that DEGs expressed at day 3 were most enriched in antigen processing and peptide or polysaccharide antigen presentation via MHC class II. Moreover, KEGG pathway analyses showed that upregulated DEGs were primarily associated with inflammatory response-related processes (CAMs, rheumatoid arthritis, and leukocyte transendothelial migration) at 5 days after fracture. These results are consistent with those of previous studies where aging was shown to affect fracture healing via a chronic increase in proinflammatory status; the notion of “inflamm-aging” has been used to describe this status in the elderly [26]. This elevated inflammatory status is associated with poorer fracture healing outcomes [27–29]; however, the mechanism remains unknown. Greater knowledge of mechanisms of this inflammatory process can be applied to positively impact fracture healing.

MSC differentiation is another key process in fracture healing, and understanding the mechanisms leading to delayed cell differentiation is important for understanding the cause of reduced skeletal regeneration with age [30, 31]. Here KEGG pathway, GO enrichment, and PPI network analyses demonstrated that the Wnt signaling pathway was downregulated at 3 days after fracture. The Wnt signaling pathway plays major roles in both skeletal development and homeostasis [32–34]. In early pluripotent MSCs, Wnt/*β*-catenin signaling must be precisely regulated to facilitate the differentiation of osteoblasts; dysregulation of this pathway alters the normal bone healing response [35, 36]. In aged animals, a reduction in MSC osteoblast differentiation ability or preferential differentiation into adipocytes may be attributable to the downregulation of the Wnt signaling pathway. Therefore, pharmacologic agents that target this pathway can yield effective therapies to improve bone repair in the elderly.

KEGG pathway enrichment and PPI network analysis revealed that the downregulated DEGs were primarily related to vascularization-associated functions (platelet activation, hematopoietic cell lineage, and VEGF signaling pathway) at 5 days after fracture. Vascularization is a complex process that is essential for successful bone repair [7]. Decreased vascular system function is likely to decrease the amount of oxygen present at the fracture site, impair nutrient exchange, and potentially cause problems during the recruitment of cells to the fracture site [7, 16]. These molecular changes may contribute to delayed fracture healing in the elderly relative to that in juveniles.

Notably, DEGs at day 5 after fracture were primarily enriched in synaptic-related functions. The exact role of sensory nerves in fracture healing remains elusive [37–39] because few studies have focused on age-related changes within the central nervous system or sensory nerves in bone repair regulation. Such synaptic alterations warrant further investigation.

One limitation of the present study is that the analyzed raw dataset did not include transcriptome data from 2, 3, 4, or 5 weeks following fracture, limiting conclusions about changes in molecular processes after bone fracture over time. Taken together, our findings demonstrate that elevated inflammatory status, a dysregulated Wnt signaling pathway, and the downregulation of vascularization-associated functions and synaptic-related processes play essential roles in fracture healing in the elderly. Further studies are required to determine the mechanisms by which these molecular events affect fracture healing.

## Figures and Tables

**Table 1:** GO terms enriched by DEGs at three time-points following bone fracture.

Category	Term	Description	P-value
Old vs young (day 1)			
	GO:0030199	collagen fibril organization	8.90E-09
	GO:0001501	skeletal system development	3.32E-06
	GO:0009888	tissue development	5.61E-06
	GO:0051241	negative regulation of multicellular organismal process	6.96E-06
	GO:0010721	negative regulation of cell development	2.37E-05
	GO:0009887	animal organ morphogenesis	2.37E-05
	GO:0060343	trabecula formation	1.04E-04
	GO:0060346	bone trabecula formation	1.08E-04
	GO:0071229	cellular response to acid chemical	1.36E-04
	GO:0001101	response to acid chemical	1.61E-04
Old vs young (day 3)			
	GO:0002504	antigen processing and presentation of peptide or polysaccharide antigen via MHC class II	5.58E-07
	GO:0071805	potassium ion transmembrane transport	7.41E-07
	GO:0071804	cellular potassium ion transport	7.41E-07
	GO:0055085	transmembrane transport	1.83E-05
	GO:0015672	monovalent inorganic cation transport	1.29E-04
	GO:1990118	sodium ion import into cell	1.77E-04
	GO:0010876	lipid localization	1.91E-04
	GO:2000054	negative regulation of Wnt signaling pathway involved in dorsal/ventral axis	1.94E-04
	GO:0034113	heterotypic cell-cell adhesion	2.40E-04
	GO:0034220	ion transmembrane transport	2.59E-04
Old vs young (day 5)			
	GO:0010951	negative regulation of endopeptidase activity	1.61E-08
	GO:0098916	anterograde trans-synaptic signaling	5.80E-07
	GO:0099537	trans-synaptic signaling	5.98E-07
	GO:0099536	synaptic signaling	7.67E-07
	GO:0006950	response to stress	1.15E-06
	GO:0051346	negative regulation of hydrolase activity	2.41E-06
	GO:0007270	neuron-neuron synaptic transmission	1.00E-05
	GO:0006955	immune response	1.81E-05
	GO:0006508	proteolysis	2.27E-05
	GO:0007268	chemical synaptic transmission	4.16E-05

GO, gene ontology; DEGs, differentially expressed genes.

## Data Availability

The original data used to support the findings of this study are available at GEO dataset (GSE17825).
